# Attentional Control and Interpretation of Facial Expression after Oxytocin Administration to Typically Developed Male Adults

**DOI:** 10.1371/journal.pone.0116918

**Published:** 2015-02-06

**Authors:** Tetsu Hirosawa, Mitsuru Kikuchi, Eiichi Okumura, Yuko Yoshimura, Hirotoshi Hiraishi, Toshio Munesue, Natsumi Takesaki, Naoki Furutani, Yasuki Ono, Haruhiro Higashida, Yoshio Minabe

**Affiliations:** 1 Department of Psychiatry and Neurobiology, Graduate School of Medical Science, Kanazawa University, Kanazawa, Japan; 2 Research Center for Child Mental Development, Kanazawa University, Kanazawa, Japan; 3 Department of Epileptology, Tohoku University School of Medicine, Sendai, Miyagi, Japan; Hamamatsu University School of Medicine, JAPAN

## Abstract

Deficits in attentional-inhibitory control have been reported to correlate to anger, hostility, and aggressive behavior; therefore, inhibitory control appears to play an important role in prosocial behavior. Moreover, recent studies have demonstrated that oxytocin (OT) exerts a prosocial effect (e.g., decreasing negative behaviors, such as aggression) on humans. However, it is unknown whether the positively valenced effect of OT on sociality is associated with enhanced attentional-inhibitory control. In the present study, we hypothesized that OT enhances attentional-inhibitory control and that the positively valenced effect of OT on social cognition is associated with enhanced attentional-inhibitory control. In a single-blind, placebo-controlled crossover trial, we tested this hypothesis using 20 healthy male volunteers. We considered a decrease in the hostility detection ratio, which reflects the positively valenced interpretation of other individuals’ facial expressions, to be an index of the positively valenced effects of OT (we reused the results of our previously published study). As a measure of attentional-inhibitory control, we employed a modified version of the flanker task (i.e., a shorter conflict duration indicated higher inhibitory control). These results failed to demonstrate any significant behavioral effects of OT (i.e., neither a positively valenced effect on facial cognition nor an effect on attentional-inhibitory control). However, the enhancement of attentional-inhibitory control after OT administration significantly correlated to the positively valenced effects on the interpretation of uncertain facial cognition (i.e., neutral and ambiguous facial expressions).

## Introduction

Over the past decade, research in various fields has revealed that oxytocin (OT) plays an important role in social interactions; its activity extends far beyond its previously documented effects on female reproduction [1–4]. Indeed, the prosocial effect of OT is one of the most attractive topics of social brain science. Recent human studies have demonstrated that administration of OT facilitates temporary attachments between strangers and increased trust, reciprocity, and generosity [1,5–8]. Intriguingly, it has been reported that OT positively valences subjects’ impressions of others’ faces [9] and optimally enhances paternal sensitivity to a child (e.g., reduces hostility) [10]. However, those prosocial effects of OT are subtle, and a minority of these published studies reported the opposite results. For example, some studies reported antisocial effects of OT, such as increased feelings of envy [11], mistrust [12], attachment insecurity [13], or outgroup derogation [14]. Taken together, a recent review proposed that the effect of oxytocin is not simply a prosocial, but rather OT exerts its prosocial effect under the constraint of inter-individual factors [15].

Several studies have suggested that aggressive (i.e., antisocial) personality traits are associated with lower attentional and/or inhibitory control. For example, Sellbom demonstrated that the traits of impulsivity, aggression and alienation were associated with lower attentional-inhibitory control as measured by the flanker task [16]. Oosterlaan et al. and Hughes et al. demonstrated in a meta-analysis that aggressive behavior (i.e., antisocial behavior) is associated with impairments in inhibitory control [17,18] and that this relationship persists over time from school age to adulthood [19,20]. Furthermore, a higher performance on theory-of-mind tasks (a prosocial characteristic) is associated with a higher performance on conflict inhibition tasks, independent of age [21,22]. These previous studies suggested an association (i.e., a positive correlation) between higher attentional control and prosociality. Although evidence of the prosocial effects of OT has accumulated in recent years, the effect of OT on attentional-inhibitory control has yet to be reported.

Recent studies have suggested that the medial prefrontal cortex (mPFC) plays an important role in social behavior [23–25]. Intriguingly, a recent study demonstrated that the effects of OT on social cognition are associated with enhanced brain coordination within the mPFC [26]. Given that the mPFC is a center of attentional-inhibition control [27,28], the effect of OT on positively valenced social cognition may be associated with enhanced attentional-inhibitory control.

Based on these findings, in the present study, we hypothesized that OT enhances attentional-inhibitory control and that the positively valenced effect of OT on social cognition is associated with enhanced attentional-inhibitory control. As an indicator of the effect of OT on social cognition, we utilized the results from our previously published placebo-controlled study [29]. In our previous study, 148 images of a variety of facial emotional expressions (e.g., happiness, anger, ambiguity, or neutrality) were presented to the study participants, and the participants reported whether they detected hostility in each facial expression. We defined the hostility detection ratio as the percentage of hostile responses among all responses to forced-choice questions (“feel hostility” or “do not feel hostility”). We regarded a decrease in the hostility detection ratio as a positively valenced interpretation of the interpersonal facial cognition. Based on the results of our previous study, we failed to demonstrate a significant positively valenced effect of OT on interpersonal facial cognition (compared to placebo) [29]. In our previous study, immediately after the hostility detection test (which lasted approx. 10 min), 19 healthy male volunteers also completed an eye-gaze version of the Eriksen flanker task (which lasted approx. seven min) [30]. We did not discuss the results of the Eriksen flanker task in our previous published report because that study focused on the modulation of the positively valenced effect of OT on interpersonal facial cognition by emotional and other characteristics of the individuals. In the present study, we focused on the enhancing effect of OT on attentional-inhibitory control and the association between the positively valenced effect of OT on interpersonal facial cognition and enhanced attentional-inhibitory control. This study is the first to demonstrate this relationship. Notably, this manuscript is based on our previously published research [29]. Specifically, the result of paradigm 1 has already been published, and the participants are identical to those of the previous report. In addition, because our previous study focused on brain activity, the participants conducted these tasks during magnetoencephalography (MEG) recording.

## Materials and Methods

### Participants

Twenty right-handed adult men participated in the experiment. The mean age of the participants was 31.4 years (range: 20–46 years). All subjects were native Japanese and had no previous or existing psychiatric, neurological, or medical disorder. The subjects were screened using a Structured Clinical Interview for DSM-IV-TR Axis I Disorders, Non-Patient edition (SCID-I/NP) diagnosis to exclude any personal history of psychiatric illness. The subjects were not on any medication for at least 6 weeks prior to the experiment, and the subjects reported a normal sleep/wake cycle. Written informed consent was obtained prior to enrollment in the study. The participants are the same as those from our previous study [29]. The Ethics Committee of Kanazawa University Hospital approved the methods and procedures used in this study, which was performed in accordance with the Declaration of Helsinki.

### Experimental design

**Fig 1:**
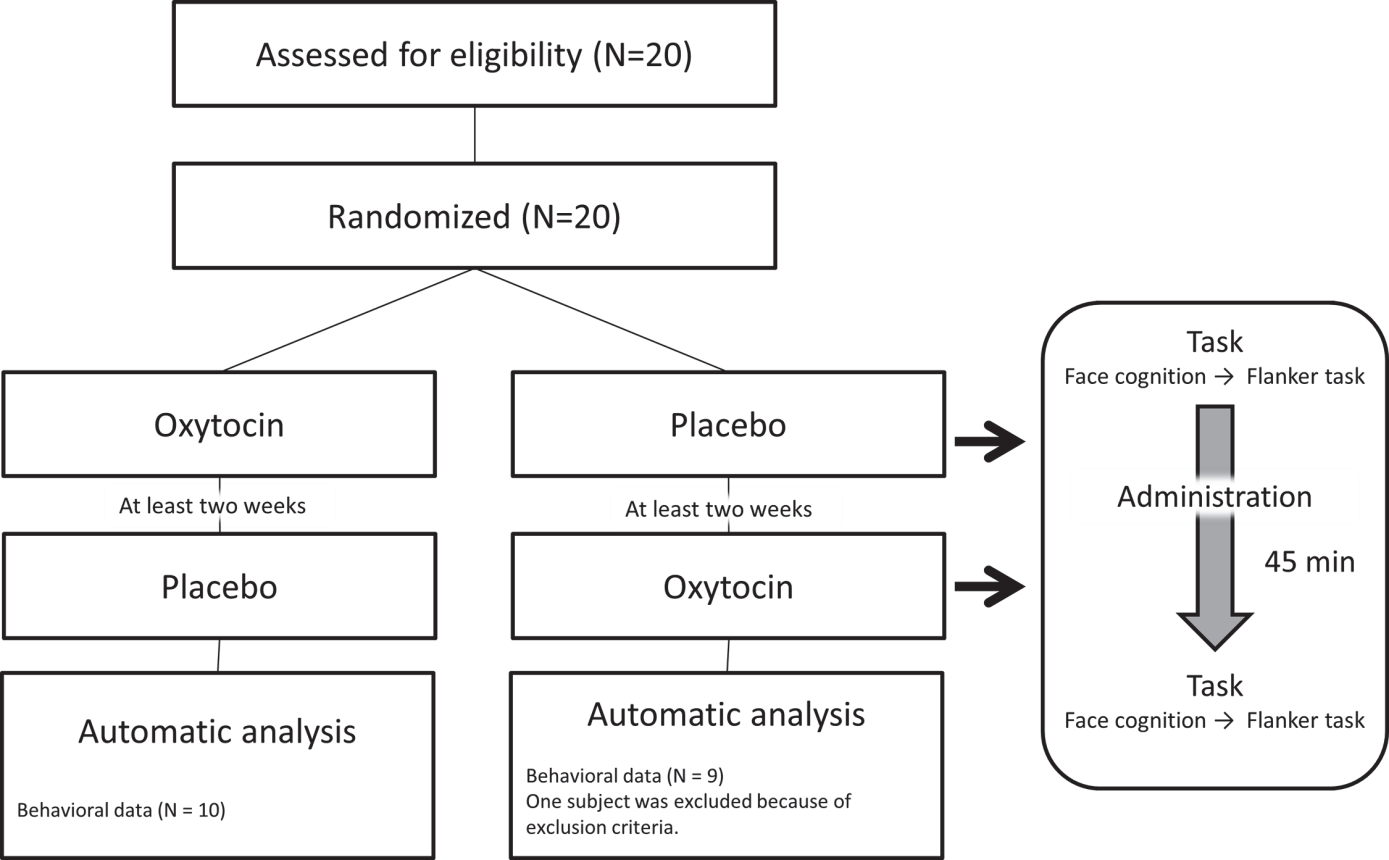
Study design. Twenty recruited participants were randomly assigned to receive either OT or placebo during the first trial. After an interval of at least 2 weeks, the second trial was conducted. The order of the two conditions (i.e., OT or placebo) was counterbalanced across subjects. Each experimental condition included two visual tasks (the interpretation of emotional facial expressions and the flanker task), and the participants conducted these different tasks twice under each condition (i.e., before and after administration). We excluded one subject from statistical analysis because of the exclusion criteria.

The experimental sessions were conducted according to a single-blind, placebo-controlled, within-subject, crossover design using an interval of at least two weeks. The mean (± standard deviation; SD) of the interval between OT and placebo administration was 73.8 (± 56.8) days. The order of the two treatment conditions (OT or placebo) was counterbalanced across subjects by random selection. The participants were randomly assigned to either the experimental condition, in which they received a single intranasal dose of 24 IU OT (Syntocinon; Novartis, Basel, Switzerland), or the placebo control condition. During each session, the participants completed the facial expression recognition task (paradigm 1) and the following flanker task (paradigm 2) prior to OT or placebo administration. Then, consistent with the published pharmacokinetics of OT [31], 45 minutes after drug or placebo administration, the participants repeated the same tasks. Thus, during each session (i.e., OT or placebo), the participants completed each task twice (before and after administration) (Fig. 1).

### Visual task procedures

The study subjects lay supine on a bed in the dark facing a tilted white screen that measured 24 × 16 cm and that was fixed above the bed. Using a video projector (PG-B10S; Sharp, Osaka, Japan), a computer projected an image onto the screen above the head of the bed at a refresh rate of 60 Hz. The distance from the subject’s nasion to the center of the screen was approximately 30 cm. Therefore, the visual angle of the image projected on the screen was approximately 42° × 34°. The visual tasks were generated using the software package SuperLab 4.0 (Cedrus, San Pedro, CA, USA).

### Paradigm 1

Paradigm 1 was designed to investigate the effect of OT on the interpretation of facial condition. The details of this paradigm and our results were reported in our previous study [29]. A total of 148 images from four categories (angry, happy, neutral, and ambiguous) of facial expression were presented in random order. After the onset of each image presentation, the participants were instructed to judge whether they sensed hostility in the face shown by pressing the appropriate button on a two-button device. We defined the hostility detection ratio as the percentage of hostile responses among all responses to forced-choice questions (“feel hostility” or “do not feel hostility”). We regarded a decrease in the hostility detection ratio as a positively valenced interpretation of the facial condition. To evaluate OT effect on this interpretation, we calculated the placebo-subtracted changes in the hostility detection ratio (i.e., OT [post—pre]—placebo [post—pre]). Paradigm 1 required approximately 10 min to complete. All participants were unfamiliar with the faces used in this task.

### Paradigm 2

**Fig 2:**
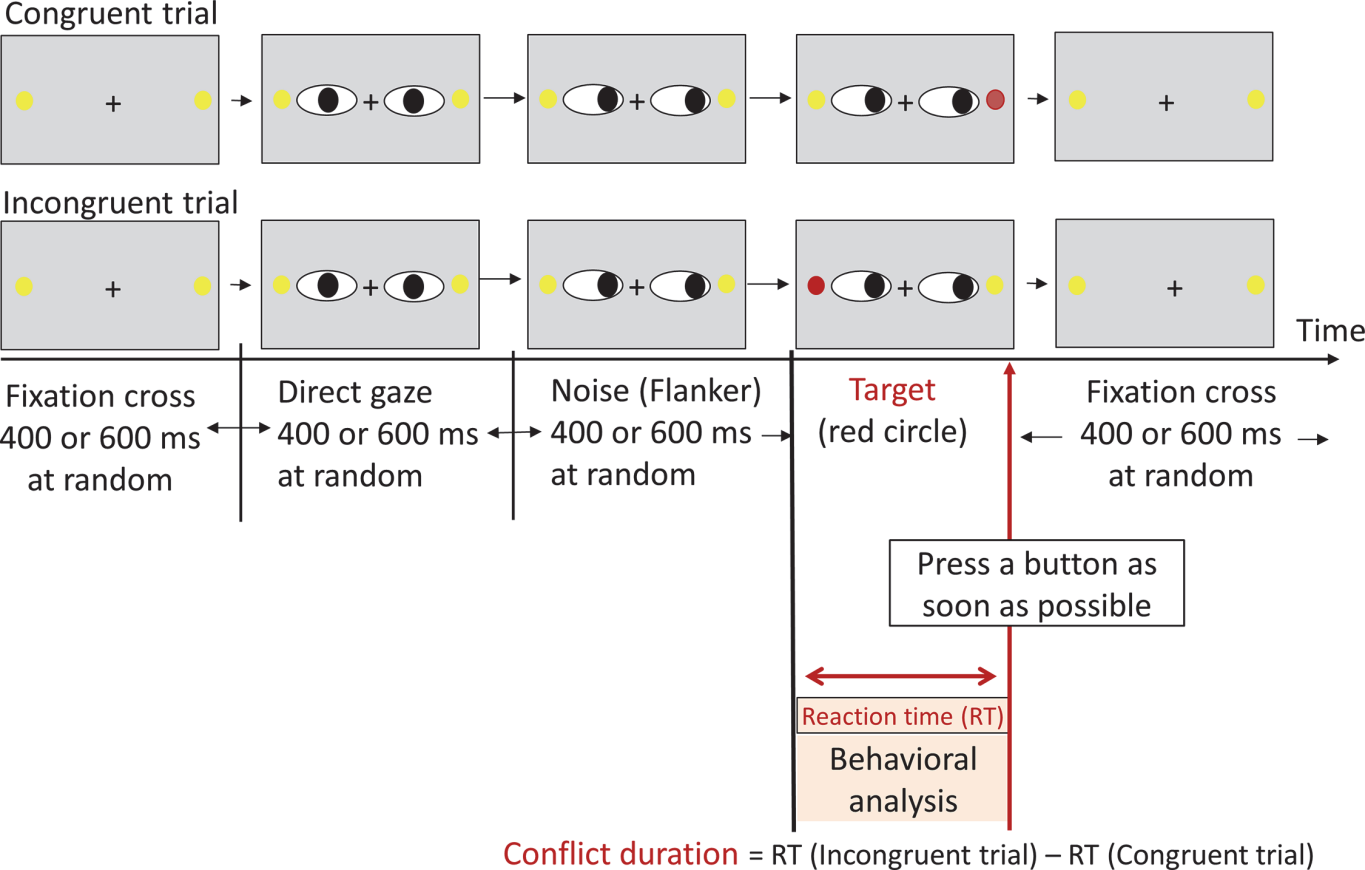
Flanker task paradigm. Each trial involved both eyes and two dots. The trial began with a fixation cross. After a random duration of 400 or 600 ms, lateral eye movements were presented as flanker distractors. Then, a color alteration of the dot followed after a random duration of 400 or 600 ms. The target stimulus was a red dot, which vanished immediately after the participant’s response (i.e., button pressing). Finally, a fixation cross was presented again for 400 or 600 ms before the next trial. A total of 148 trials (74 congruent trials and 74 incongruent trials) were presented in a randomized order. The total time of the task was approximately 7 min.

After paradigm 1 was completed, paradigm 2 was initiated. Paradigm 2 was designed to investigate the effect of OT on attentional-inhibitory control. Paradigm 2 was a modification of the speeded flanker task [32]. Before the initiation of the task, the participants were instructed to respond to the target dot at maximal accuracy and speed by pressing a button using the thumb of the corresponding hand (left/right). Each trial used both eyes and was initiated as a fixation cross and the presentation of two dots for a random duration of 400 or 600 ms. Lateral eye movements as flanker distractors (due to noise stimuli) were followed by color alteration of the dot (i.e., target stimuli) after 400 or 600 ms. The target stimuli were removed immediately after the participant’s response (i.e., button pressing). Finally, a fixation cross was presented for 400 or 600 ms before the next trial. No instructions regarding the correction of erroneous responses were provided. The participants were required to fix their gaze on the central cross to avoid eye movements throughout the experimental session (Fig. 2). A total of 148 trials was performed. This task required approx. 7 min. For each subject, the trials in which the reaction time (RT) exceeded two SDs from the subject’s mean value for each response type were discarded from the analysis as outliers. The RTs for the congruent trial were subtracted from those for the incongruent trial, and we used these values (i.e., conflict duration) as indices of attentional-inhibitory control. As in paradigm 1, we calculated the placebo-subtracted changes (i.e., OT [post—pre]—placebo [post—pre]) to evaluate the effect of OT on the RT.

### Statistics

Via two-way analysis of variance (ANOVA), all of the factors were subjected to within-subjects analyses of the treatment effect (pre- vs. post-treatment) and the drug effect (OT vs. placebo). ANOVA (treatment × drug) was performed to evaluate the behavioral changes in the hostility detection ratio (for each emotional face condition) or in conflict duration. Statistical significance was defined as *P* < 0.05.

During the pre-treatment periods (i.e., baseline), to determine whether positively valenced facial cognition was associated with higher attentional-inhibitory control, we performed a simple correlation analysis using the Spearman rank correlation coefficient between the results of the paradigms (i.e., the hostility detection ratio in paradigm 1 and the conflict duration in paradigm 2 during the pre-treatment periods) for the OT and placebo conditions. Given that the relationship between positively valenced facial cognition and higher attentional-inhibitory control would be subtle and not sufficiently strong to appear in easily identifiable facial cognitions (i.e., angry or happy expressions), we used the mean values of the hostility detection ratio for only the uncertain facial expressions (i.e., neutral and ambiguous conditions).

To test our hypothesis that the positively valenced effect of OT on facial condition is associated with OT-induced enhancements in attentional-inhibitory control, we analyzed the significance of the relationships in the next procedure. First, the pre-treatment values were subtracted from the post-treatment values (i.e., post—pre). Then, we performed a simple correlation analysis using the Spearman rank correlation coefficient between the subtracted variables (i.e., post—pre) of the two paradigms (i.e., the hostility detection ratio in paradigm 1 and the conflict duration in paradigm 2) for the OT and placebo conditions. Second, the placebo treatment values (i.e., post—pre) were subtracted from the OT treatment values (i.e., post—pre) to exclude a placebo effect. Finally, using these subtracted variables (i.e., OT [post—pre]—placebo [post—pre]), we performed a simple correlation analysis using the Spearman rank correlation coefficient between the two paradigms (i.e., the hostility detection ratio in paradigm 1 and the conflict duration in paradigm 2). Given that the association between the effects of OT on the positively valenced interpretation of facial condition and enhanced attentional-inhibitory control would be subtle and not sufficiently strong to alter judgments about easily identifiable facial expressions (i.e., angry or happy expressions), we hypothesized that the OT-induced changes in attentional-inhibitory control positively correlate to the mean change in the positively valenced interpretation of the uncertain facial expressions (i.e., neutral and ambiguous conditions). Statistical significance was defined as *P* < 0.05.

## Results

As shown in Fig. 1, the experimental sessions were conducted according to a single-blind, placebo-controlled, within-subject, crossover design using an interval of at least 2 weeks. The order of the two conditions (OT or placebo) was counterbalanced across subjects by random selection. We excluded one subject from the behavioral study because he met the exclusion criteria of our previous neurophysiological study (i.e., unrecoverable magnetic noise caused by a dental bridge) [29]. He was removed from the study at the end of paradigm 1; therefore, he did not complete paradigm 2. Thus, the subjects for statistical analysis in the present study consisted of 19 males (10 who began under the OT condition and nine who began under the placebo condition). On the flanker task, the mean values (SDs) of the RT values for the congruent trial were 320 (66) ms for correct responses and 250 (133) ms for incorrect responses. The mean values (SDs) of the RT values for the incongruent trial were 330 (66) ms for correct responses and 230 (69) ms for incorrect responses. The mean values (SDs) of the correct response ratio were 99.4 (1.0) % for the congruent trial and 99.8 (0.2) % for the incongruent trial.

### Two-way analysis of variance (ANOVA) (treatment × drug) of the hostility detection ratio

To determine whether OT induced changes in the hostility detection ratio, two-way ANOVA (treatment × drug) was performed. There was no significant drug effect on any emotional condition (i.e., happiness [df = (1,18), F = 0.15, *P* > 0.05], anger [df = (1,18), F = 0.94, *P* > 0.05], ambiguity [df = (1,18), F = 1.61, *P* > 0.05], or neutral condition [df = (1,18), F = 2.23, *P* > 0.05]), and there was no significant interaction between these two factors under any emotional condition (i.e., happiness [df = (1,18), F = 0.89, *P* > 0.05], anger [df = (1,18), F = 0.94, *P* > 0.05], ambiguity [df = (1,18), F = 0.17, *P* > 0.05], or neutral condition [df = (1,18), F = 0.02, *P* > 0.05]). Therefore, we failed to demonstrate an OT-related effect on the hostility detection ratio.

**Fig 3:**
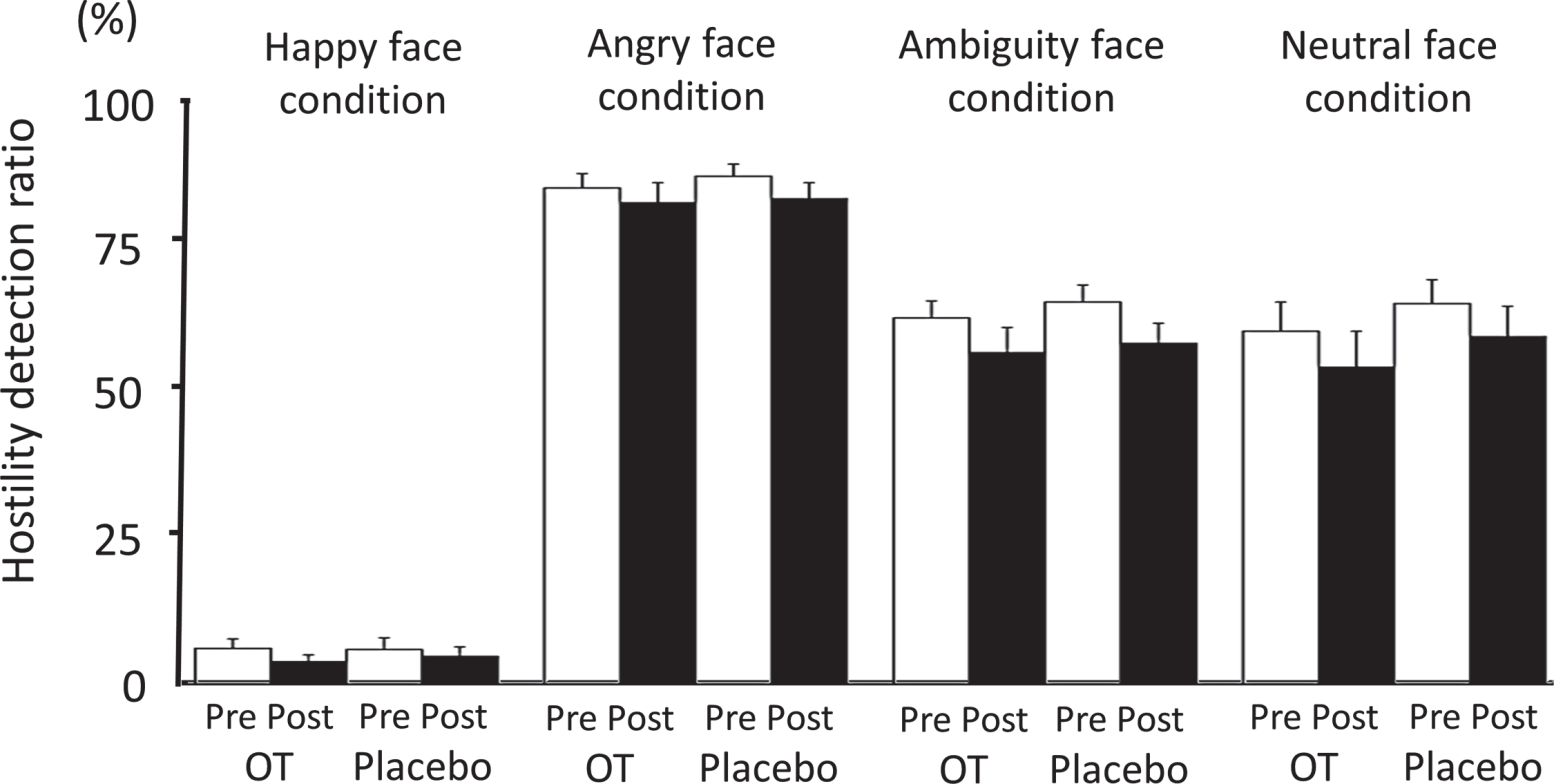
The hostility detection ratio (a lower value indicates a positively valenced interpretation of the facial expression) pre- (white bar) and post-treatment (black bar) with OT or placebo. A, happy face condition. B, angry face condition. C, ambiguous face condition. D, neutral face condition. Two-way ANOVA (treatment × drug) revealed significant treatment effects (i.e., order effects) under the ambiguous and neutral conditions but not under the happy or angry condition. We failed to detect an OT-related effect on the hostility detection ratio under any condition. The error bars represent 1 standard error.

There were significant treatment effects on the ambiguity (df = (1,18), F = 15.02, *P* = 0.001) and neutral condition (df = (1,18), F = 9.79, *P* = 0.006) (Fig. 3) but not on the happiness (df = (1,18), F = 3.50, *P* > 0.05) or anger condition (df = (1,18), F = 4.17, *P* > 0.05).

### Two-way analysis of variance (ANOVA) (treatment × drug) of attentional-inhibitory control

**Fig 4:**
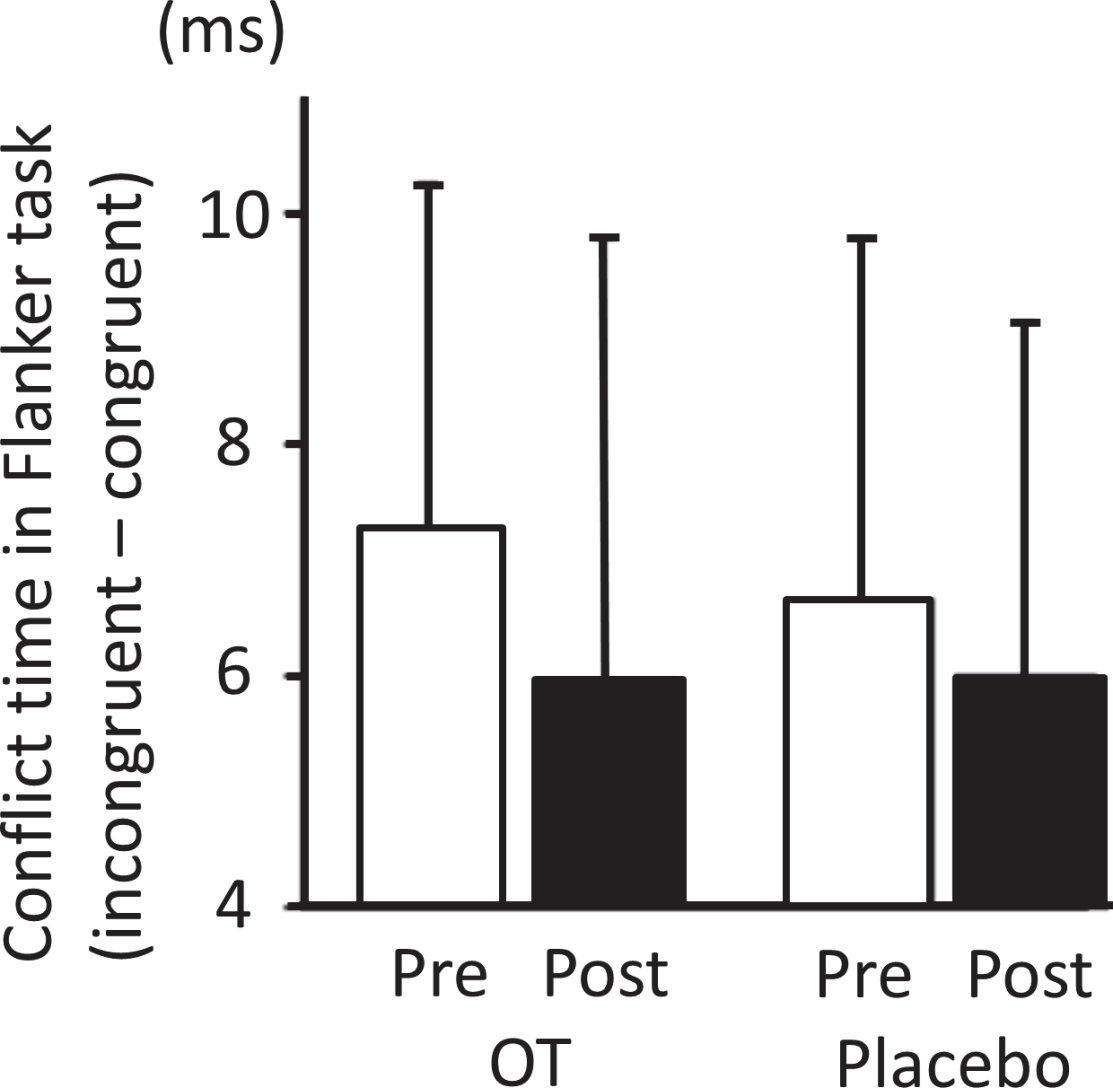
Conflict duration on the flanker task (i.e., reaction time for each incongruent trial–reaction time for each congruent trial) pre- (white bar) and post-treatment (black bar) with OT or placebo. Two-way ANOVA (treatment × drug) failed to demonstrate any drug or treatment effects, and no significant interaction between these two factors was detected. Therefore, we failed to demonstrate any OT-related effect on attentional-inhibitory control. The error bars represent 1 standard error.

To determine whether OT induced changes in attentional-inhibitory control (i.e., conflict duration), two-way ANOVA (treatment × drug) was performed. No significant drug effect (df = (1,18), F = 0.01, *P* > 0.05), treatment effect (df = (1,18), F = 0.24, *P* > 0.05), or interaction between these two factors (df = (1,18), F = 0.02, *P* > 0.05). Therefore, we failed to demonstrate an OT-related effect on attentional-inhibitory control (Fig. 4).

### Facial cognition and attentional-inhibitory control during the pre-treatment baseline period

During the pre-treatment period (i.e., baseline), Spearman rank correlation revealed significant correlations between the results of the two paradigms (i.e., the hostility detection ratio in paradigm 1 and the conflict duration in paradigm 2 during the pre-treatment period) under OT (ρ = 0.49, *P* = 0.040) and placebo conditions (ρ = 0.64, *P* = 0.006).

### Correlation between the positively valenced effects of OT on interpretation of the facial expression and the OT-induced changes in attentional-inhibitory control

For the treatment effect (i.e., post—pre), Spearman rank correlation between the subtracted variables of the two paradigms (i.e., the hostility detection ratio in paradigm 1 and the conflict duration in paradigm 2) failed to demonstrate any significance for the OT (ρ = 0.39, *P* > 0.05) or placebo (ρ = 0.06, *P* > 0.05) condition.

**Fig 5:**
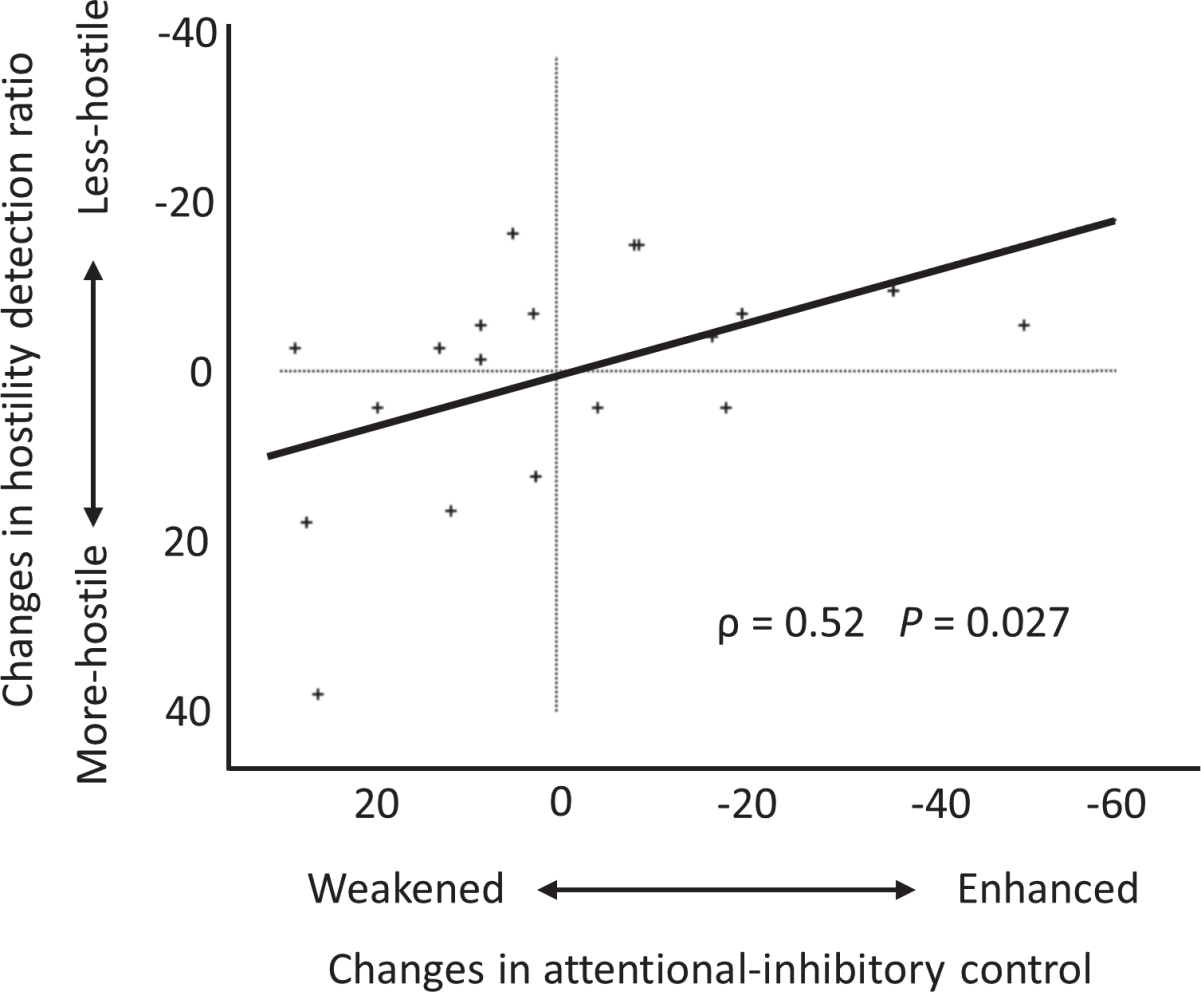
Scatter plot of the OT-induced changes on the facial emotion recognition task and on the flanker task. The vertical axis represents the OT-induced changes (i.e., OT [post—pre]—placebo [post—pre]) in the interpretation of facial expression for the combined uncertain (i.e., neutral and ambiguous) conditions. The horizontal axis represents the OT-induced changes (i.e., OT [post—pre]—placebo [post—pre]) in conflict duration on the flanker task. Notably, the change to less-hostile facial cognition was associated with enhanced attentional-inhibitory control after OT administration. The solid line denotes the regression line.

For the placebo-subtracted treatment effect (i.e., OT [post—pre]—placebo [post—pre]), Spearman rank correlation demonstrated significant correlations between the changes in the interpretation of facial expressions and the changes in attentional-inhibitory control (ρ = 0.52, *P* = 0.027; see Fig. 5). The interpretation of less hostile facial condition was associated with enhanced attentional-inhibitory control after OT administration.

## Discussion

We examined whether OT enhances positively valenced social cognition and attentional-inhibitory control. The results of the two-way ANOVA (treatment × drug) of the hostility detection ratio did not reveal any significant effects of the drug (i.e., OT vs. placebo) or any drug-related interactions under each emotional face condition. The results of the two-way ANOVA (treatment × drug) of attentional-inhibitory control (i.e., conflict duration) did not reveal any significant effects of the drug (i.e., OT vs. placebo) or any drug-related interactions. These findings suggest that the effect of OT was small or varied among individuals; therefore, its effect did not reach statistical significance for the entire group. However, we observed a treatment effect (i.e., an order effect) on the hostility detection ratio under the ambiguity and neutral conditions. A habituation effect may explain this result. To detect subtle effects of OT on the interpretations of both facial expression and attentional-inhibitory control, further studies using larger sample sizes might be required.

Next, we examined whether the effect of OT on social cognition is associated with changes in attentional-inhibitory control. First, a Spearman rank correlation analysis of the baseline condition (i.e., pre-treatment) revealed significant correlation between the hostility detection ratio in paradigm 1 and the conflict duration in paradigm 2. This finding suggests that individuals with higher attentional-inhibitory control tend to have a higher threshold for hostility detection. This is consistent with previous research [33, 34], suggesting that we could regard our tasks as valid. Second, the pre-treatment values were subtracted from the post-treatment values (i.e., post—pre), and we performed a simple correlation analysis between the subtracted variables (i.e., post—pre) of the two paradigms (i.e., the hostility detection ratio in paradigm 1 and the conflict duration in paradigm 2) for the OT and placebo conditions, respectively. Although the *P* value did not reach the statistical significance level, there was a trend toward a positive correlation for the OT condition (ρ = 0.39, *P* < 0.1) but not for the placebo condition (ρ = 0.06). Third, to disconfirm the contributions of placebo and habituation effects on these correlation analyses, the placebo treatment values (i.e., post—pre) were subtracted from the OT treatment values (i.e., post—pre). Spearman rank correlations for the placebo-subtracted treatment effect (i.e., OT [post—pre]—placebo [post—pre]) demonstrated significant correlations between the changes in the interpretation of facial expressions and the changes in attentional-inhibitory control (ρ = 0.52, *P* = 0.027). In other words, the positively valenced effect of OT on the interpretation of facial cognition significantly correlated with the enhancement in attentional-inhibitory control. These findings suggest that individuals who exhibit a beneficial effect of OT on attentional-inhibitory control tend to interpret others’ uncertain facial expressions as being “less hostile.”

The measurement of attentional-inhibitory control (i.e., the flanker task) that we performed in the present study represents one aspect of executive function, and our results also support the possibility that greater executive function helps people to be prosocial. Consistent with our findings, some previous studies have demonstrated that people with lower executive function tend to exhibit angry, hostile, and aggressive behavior (i.e., antisocial behavior) [33] and that people with antisocial personality disorder display impairments in executive function [34]. Furthermore, possessing a theory of mind is thought to contribute to the development of a prosocial orientation [35], and previous studies have demonstrated robust correlations between performance on theory of mind tasks and performance on executive function tasks (including conflict inhibition tasks), independent of age and intelligence [21,22,36]. These previous studies, in conjunction with our present results, suggest that executive function (including attentional-inhibitory control) is a characteristic that produces prosocial behavior.

Given that serotonergic neurons densely express OT receptors [37], enhanced serotonergic transmission may be a common mechanism that underlies the association between the prosocial effects of OT and enhanced executive function. Several recent behavioral studies have suggested that the serotonin system affects both conflict inhibition tasks [38] and the interpretation of facial expressions [39]. Recent studies have suggested that the serotonin system affects the interpretation of facial expressions via PFC-amygdala circuits [40]; these regions also play an important role in conflict inhibition [28,41–43]. Consistent with these studies of the serotonin system, recent studies of the OT system demonstrated that OT exerts its prosocial effect on facial emotion recognition via an enhancement of activity in the amygdala [29,44] and that OT enhances the resting-state connectivity of PFC-amygdala circuits [45]. Taken together, these results suggest that OT exerts its prosocial effect and enhances inhibitory control via the serotonergic system involving PFC-amygdala circuits.

The present study contains some limitations. First, the sample size was small, and the subjects were exclusively male. It will be important to replicate these findings using a larger sample that includes both genders and a wider age range. Second, because the substances were administered in a single-blind manner (i.e., the experimenter knew the composition of the groups, but the participants did not), we cannot firmly exclude the possibility that the experimenter involuntarily influenced the findings. However, this possibility is unlikely because (a) verbal contact with the experimenter was limited (all instructions during the task were provided by the computer), and (b) the instructions were fully standardized. Third, because the participants repeated the same task (i.e., before and after administration of OT/placebo), they might have adapted to the Flanker task. A relatively strong effect of adaptation or a placebo effect might obscure any relatively weak differences between OT and placebo. Fourth, to detect the subtle effects of OT, a more implicit method might be required. In our facial emotion recognition task, the participants explicitly determined whether the facial expression was hostile. Considering the relatively weak difference between OT and placebo, a more implicit method might be suitable for detecting any subtle differences.

## Conclusions

Our results suggest that the various effects of OT on the interpretation of facial cognition are large and are associated with the enhancement of attentional-inhibitory control. These findings may enable deeper insight into the existing and emerging experimental data regarding the prosocial effects of OT on facial cognition.
